# Perceived organizational climate and turnover intention among young nurses from a humanistic care perspective: the mediating role of work engagement

**DOI:** 10.3389/fpsyt.2026.1824434

**Published:** 2026-06-11

**Authors:** Kaili Fan, Jingwen Zhang, Yixi Huang, Xue Li, Xi Zhou, Huiyun Yang

**Affiliations:** 1 Department of Nursing Administration, The Second Affiliated Hospital of Xi’an Jiaotong University, Xi’an, China; 2 Nursing Faculty of Medical Department, Xi’an Jiaotong University, Xi’an, China; 3 Health Management Department, The Second Affiliated Hospital of Xi’an Jiaotong University, Xi’an, China

**Keywords:** humanistic care, mediating effect, perceived organizational climate, turnover intention, work engagement, young nurse

## Abstract

**Background:**

The high turnover of young nurses poses a significant challenge to the stability of healthcare systems worldwide. While the relationships between perceived organizational climate, work engagement, and turnover intention are established, there is a lack of research integrating a humanistic care management perspective to elucidate the specific psychological mechanisms among young nurses at their uniquely vulnerable career stage. Drawing upon this contextual lens, this study aims to evaluate the status of perceived organizational climate, work engagement, and turnover intention among young nurses, and to further explore the potential mediating role of work engagement in the relationship between perceived organizational climate and turnover intention.

**Methods:**

A cross-sectional study was conducted from July to September 2025, surveying 366 young nurses from a tertiary Grade-A hospital in Shaanxi Province, China, using convenience sampling. Data were collected using a general information questionnaire, the Utrecht Work Engagement Scale, the Organizational Climate Questionnaire, and the Turnover Intention Questionnaire. A Structural Equation Model was employed to analyze the mediating effect.

**Results:**

The findings revealed significant interrelationships among perceived organizational climate, work engagement, and turnover intention. Work engagement was found to partially mediate the relationship between perceived organizational climate and turnover intention during young nurses, explaining 34.78% of the variance.

**Conclusion:**

These findings suggest that organizational climate functions as a critical job resource that may buffer turnover intention by fostering higher levels of work engagement. To maintain workforce stability, nursing managers should integrate humanistic care into organizational policies to cultivate a supportive environment. However, due to the cross-sectional design and convenience sampling from a single institution, causal inferences should be made with caution, and the generalizability of the findings may be limited.

## 1 Introduction

The nursing workforce is currently facing a significant global crisis, with high turnover rates posing a major threat to the quality of patient care and the sustainability of healthcare systems (1). This issue is particularly pronounced in China, where the rapid expansion of healthcare services has placed unprecedented pressure on frontline staff (2). Article 28 of the 2022 update of the International Code of Medical Ethics mentions that medical staff should pay attention to their own health and physical and mental well-being to ensure their safe practice. The individual well-being of medical staff and the concept of caring for doctors have been paid more and more attention (3).

Theoretically, humanistic care is defined as the concern for an individual’s state of existence and the maintenance of their dignity and value, encompassing care, respect, and nurturing (4). In recent years, strengthening humanistic care in medicine has emerged as a central theme in China’s healthcare system reform (5). However, within this context, nurses, who are typically the providers of care, often find their own needs for care overlooked (6). This neglect is problematic, as the UK’s National Health Service (NHS) emphasizes that the sustainability of high-quality nursing is contingent upon a reciprocal humanistic framework that high-quality nursing necessitates the provision of humanistic care to both patients and nurses (7).

Young nurses represent the backbone of the nursing workforce. They not only undertake the majority of clinical care responsibilities but are also a high-risk group for career mobility due to their specific career stage (8, 9). Their high turnover rate poses a direct threat to the continuity and quality of healthcare services. Turnover intention, defined as the psychological state wherein an individual contemplates and plans to leave their current position (10), is particularly prevalent among young nurses. Research indicates that factors such as limited clinical experience, developing communication skills, and lower stress-coping abilities make young nurses more susceptible to developing strong turnover intentions (11).

In exploring the antecedents of turnover intention, organizational-level variables are of paramount importance. Perceived organizational climate, which refers to a nurse’s subjective perception of their work environment’s characteristics and conditions, has been identified as a key factor influencing their occupational health outcomes (12). Grounded in Noddings’ Ethics of Care, we recognize that while nurses are the primary ‘care giver’ in clinical practice, the sustainability of this role depends on the needs of the ‘care giver’ themselves being met. Therefore, a supportive organizational climate serves as the essential environment that upholds the nurses’ dignity and professional worth (13). Meanwhile, work engagement, characterized as a positive and fulfilling work-related state of mind, not only enhances nursing quality and innovative behaviors but also serves as a significant negative predictor of turnover intention (14, 15).

This study is theoretically grounded in the Job Demands-Resources (JD-R) model (16). This model posits that job demands are the negative aspects of a job that consume an individual’s energy, whereas job resources are the positive aspects that facilitate the achievement of work goals and buffer the negative impact of demands. Within the framework of this study, a positive perceived organizational climate, manifested through elements like managerial support and team cohesion can be conceptualized as a critical job resource. From a humanistic perspective, this climate represents a strategic resource allocation of socio-emotional assets that directly enhances employee well-being by fulfilling the fundamental psychological needs of nurses as caregivers (17). These resources do not merely function as passive supports, they catalyze a psychological transformation where external care is internalized into work engagement, a positive and fulfilling state of mind. Following the JD-R motivational pathway, when individuals perceive ample job resources, their level of work engagement increases. Conversely, a lack of resources can lead to diminished engagement, thereby increasing the likelihood of turnover intention (18).

Despite extensive research on nurse turnover, critical gaps remain. First, many existing studies treat organizational climate as a collection of generic administrative policies or physical conditions, often overlooking the socio-emotional and ethical dimensions that define the nursing profession (19). This is particularly problematic in the context of young nurses, whose professional identity is still in a formative and vulnerable stage. Furthermore, while the JD-R model identifies the motivational path of job resources, empirical findings regarding the strength of this path are inconsistent. Specifically, the precise motivational process through which perceived humanistic resources are internalized into work engagement remains underexplored (20). Without systematically interrogating how a “humanistic” climate fosters the vigor and dedication necessary for clinical practice, we cannot fully explain the mechanisms that buffer young nurses’ turnover intentions despite high job demands.

To address these gaps, the present study, drawing on a humanistic care perspective and integrating the JD-R model, aims to investigate the relationship between perceived organizational climate and turnover intention among young nurses, with a specific focus on examining the mediating effect of work engagement. Specifically, we position work engagement as a critical psychological bridge that explains how a care-oriented environment translates into a reduced desire to leave. By constructing and testing a structural equation model, this research seeks to elucidate the underlying mechanisms that have been underexplored in previous dyadic studies. The findings are expected to provide nursing managers with a theoretical basis and empirical evidence to develop humanistic management strategies that enhance work engagement through an improved organizational climate, thereby fostering long-term retention among young nurses.

## 2 Materials and methods

### 2.1 Study design

A cross-sectional survey design was employed. Using the collected data, a structural equation model (SEM) reflecting the study’s findings was developed.

### 2.2 Sampling and participants

From July to September 2025, young nurses from a tertiary Grade-A hospital in Shaanxi Province, China, were recruited using convenience sampling. Sample Size Calculation: According to Kendall’s sample size estimation method and the requirements for Structural Equation Modeling (SEM), the sample size should be 5 to 10 times the number of observed variables. This study included 56 observed items, yielding a minimum required sample size of 280. Factoring in a potential 20% attrition rate for invalid responses, the target sample size was at least 350 (280/0.8). The final sample of 366 participants was deemed adequate for the analysis.

Participants were included based on the following:

1. aged under 35 years.
2. registered nurses holding a valid practice license and working in a clinical capacity.
3. employed in their current department for a continuous period of at least six months.
4. provided informed consent and voluntarily agreed to participate.

The exclusion criteria are as follows:

1. individuals in nursing management positions (e.g., head nurses or above).
2. nurses on temporary rotation or advanced training programs.
3. nursing interns.
4. those unavailable during the survey period due to sick leave, personal leave, or other reasons.

These groups were excluded to ensure the sample uniquely represented frontline formal employees with stable perceptions of the perceived organizational climate.

### 2.3 Instruments

#### 2.3.1 General information questionnaire

A self-developed questionnaire collected demographic and professional data, including gender, age, marital status, educational level, professional title, work experience, and monthly income.

#### 2.3.2 Organizational climate questionnaire

The OCQ, developed by He Ye (21), is a localized scale suitable for the Chinese hospital cultural context. It comprises 37 items across six dimensions: Resource Support, Team Behavior, Managerial Support, Quality Management, Human Resource Management, and Evidence-Based Nursing Support. Items are rated on a 4-point Likert scale (1 = strongly disagree, 4 = strongly agree), with total scores ranging from 37 to 148. Higher scores indicate a more positive perceived organizational climate. The original scale demonstrated good reliability (Cronbach’s α = 0.95). In the present study, the scale’s Cronbach’s α was 0.983, indicating excellent internal consistency.

#### 2.3.3 Utrecht work engagement scale 15

The Chinese version of the UWES-15, revised by Zhang Yiwen et al. (22), was used. This scale consists of 15 items measuring three dimensions: Vigor, Dedication, and Absorption. It uses a 7-point Likert scale (0 = never, 6 = always), with total scores ranging from 0 to 90. Higher scores reflect greater work engagement. The revised scale has shown good psychometric properties (Cronbach’s α = 0.90). In this study, the Cronbach’s α for the scale was 0.963, demonstrating high internal consistency.

#### 2.3.4 Turnover intention questionnaire

A 4-item scale developed by Pei Yan (23) was used to measure turnover intention. It is rated on a 5-point Likert scale (1 = strongly disagree, 5 = strongly agree), with total scores from 4 to 20. Higher scores signify a stronger intention to leave. The original scale’s Cronbach’s α was 0.88. In the present study, the Cronbach’s α was 0.939, indicating excellent internal consistency.

### 2.4 Data collection and quality control

Data were collected via an online questionnaire platform. The online approach was selected to accommodate the nurses’ shift-work schedules and to ensure data completeness through mandatory response settings. After obtaining approval from the nursing department of the participating hospital, head nurses in each unit distributed the questionnaire link and QR code to eligible nurses. The initial page of the online survey featured a comprehensive description of the research, detailing its purpose, significance, and strictly adhering to data confidentiality principles. It explicitly emphasized the voluntary nature of participation. To ensure informed consent, participants were required to read this information and click a ‘Confirm’ button as an agreement to participate before being granted access to the questionnaire. To ensure data quality, the following measures were implemented: ① the system was configured to allow only one submission per IP address or device; ② all questions were set as mandatory to prevent missing data; and ③ submissions were monitored in real-time, and questionnaires with excessively short completion times (e.g., <300 seconds), patterned responses (e.g., selecting the same option for all items), or clear logical inconsistencies were excluded from the final analysis.

### 2.5 Statistical analysis

Data were analyzed using SPSS 25.0 and AMOS 26.0. Categorical variables, such as demographic data, were described using frequencies and percentages [n(%)]. Continuous variables, including scale scores, were described using means and standard deviations (M ± SD). Pearson correlation analysis was conducted to examine the relationships between the total scores and dimensions of perceived organizational climate, work engagement, and turnover intention.

Preliminary diagnostics confirmed univariate normality (skewness and kurtosis within ±2) and the absence of multicollinearity (VIF < 10) (24). To rigorously assess common method bias, the unmeasured latent methods factor (ULMC) was used to test the common method bias.

Finally, a Structural Equation Model (SEM) was constructed to test the mediating role of work engagement between perceived organizational climate and turnover intention. The significance of the mediating effect was verified using a bias-corrected bootstrapping method with 5,000 resamples to generate 95% confidence intervals (CI). Model fit was evaluated using the following indices: chi-square to degrees of freedom ratio (χ²/df) < 3.0 (ideal) or < 5.0 (acceptable), Root Mean Square Error of Approximation (RMSEA) < 0.08, and Goodness-of-Fit Index (GFI), Comparative Fit Index (CFI), Normed Fit Index (NFI), Incremental Fit Index (IFI), and Tucker-Lewis Index (TLI) all > 0.90. The level of statistical significance was set at α = 0.05.

## 3 Results

### 3.1 Characteristics of the participants

Among 366 young nurses, the majority of participants were female (96.4%), aged between 26 and 35 years (83.1%), married (59.3%), held a bachelor’s degree (98.4%), and were registered nurses (Nurse Practitioner title, 61.2%). Detailed demographic characteristics are presented in **Table 1**.

**Table 1:** Demographic characteristics of participants (n=366).

Variable	Category	n	%
Gender	Male	13	3.6
	Female	353	96.4
Age (years)	≤25	62	16.9
	26-35	304	83.1
Marital status	Unmarried	145	39.6
	Married	217	59.3
	Other	4	1.1
Education	Junior college	3	0.8
	Bachelor’s degree	360	98.4
	Master’s degree or above	3	0.8
Professional title	Nurse	76	20.8
	Nurse practitioner	224	61.2
	Senior nurse practitioner	66	18.0
Work experience (years)	1-5	145	39.6
	6-10	110	30.1
	>10	111	30.3
Monthly income (CNY)	≤5000	19	5.2
	5001-8000	177	48.4
	>8000	170	46.4

### 3.2 Assessment measurement model

A confirmatory factor analysis (CFA) was conducted to evaluate the measurement model, which included ten latent constructs: the six sub-dimensions of perceived organizational climate, the three sub-dimensions of work engagement, and the single dimension of turnover intention (represented by its four items). The CFA results demonstrated an acceptable fit for the measurement model: χ2/df=3.886 (<3.0), RMSEA = 0.079(<0.08), CFI = 0.969, TLI = 0.962 and RMR = 0.032. Following the confirmation of the model fit, the reliability and convergent validity were assessed. As detailed in **Table 2**.

**Table 2:** Reliability and convergent validity results of the measurement model.

Variable	Dimensions	Items	Loadings (range)	α	CR	AVE
Perceived organizational climate	Managerial support	9	0.661 - 0.873	0.939	0.940	0.636
	Human resource management	4	0.779 - 0.851	0.887	0.891	0.671
	Resource support	10	0.661 - 0.877	0.947	0.947	0.644
	Team behavior	8	0.731 - 0.899	0.945	0.948	0.696
	Quality management	4	0.844 - 0.891	0.923	0.925	0.755
	Evidence-based nursing support	2	0.875 - 0.892	0.876	0.877	0.781
Work engagement	Vigor	6	0.770 - 0.911	0.942	0.945	0.742
	Dedication	4	0.834 - 0.890	0.918	0.921	0.745
	Absorption	5	0.675 - 0.879	0.908	0.911	0.673
Turnover intention	Single factor	4	0.857 - 0.936	0.939	0.940	0.797

### 3.3 Descriptive statistics of key variables

The mean total score for turnover intention, perceived organizational climate, work engagement were 10.56 ± 3.99, 120.70 ± 21.99, 71.82 ± 22.41, respectively. Detailed scores are shown in **Table 3**.

**Table 3:** Descriptive statistics of scores for key variables (M ± SD).

Variable	Total/dimension	Total score	Item-mean score
Turnover intention		10.56 ± 3.99	2.64 ± 1.00
Perceived organizational climate	Total score	120.70 ± 21.99	3.36 ± 0.59
	Resource support	30.78 ± 6.87	3.08 ± 0.69
	Team behavior	27.44 ± 4.45	3.43 ± 0.56
	Managerial support	29.51 ± 5.62	3.28 ± 0.62
	Quality management	13.67 ± 2.30	3.42 ± 0.57
	Human resource management	12.69 ± 2.85	3.17 ± 0.71
	Evidence-based nursing support	6.62 ± 1.40	3.31 ± 0.70
Work engagement	Total score	71.82 ± 22.41	4.79 ± 1.49
	Vigor	29.90 ± 8.98	4.98 ± 1.50
	Dedication	18.51 ± 6.78	4.63 ± 1.69
	Absorption	23.42 ± 7.66	4.68 ± 1.53

### 3.4 Correlation analysis of key variables

Pearson correlation analysis revealed significant relationships among the main variables. As shown in **Table 4**, perceived organizational climate was significantly and negatively correlated with turnover intention (r = -0.647, P < 0.01). Work engagement was also significantly and negatively correlated with turnover intention (r = -0.617, P < 0.01). Furthermore, a significant positive correlation was found between perceived organizational climate and work engagement (r = 0.729, P < 0.01). The significant correlations between variables provide support for testing the mediating effect.

**Table 4:** Pearson correlation matrix for all variables and dimensions (r-values).

Variable	OC	MS	HRM	RS	TB	QM	EBNS	WE	V	D	A	TI
OC	1											
MS	.965*	1										
HRM	.879*	.827*	1									
RS	.963*	.914*	.833*	1								
TB	.936*	.879*	.762*	.851*	1							
QM	.913*	.845*	.748*	.835*	.896*	1						
EBNS	.854*	.792*	.722*	.786*	.798*	.836*	1					
WE	.729*	.704*	.647*	.699*	.685*	.654*	.635*	1				
V	.712*	.688*	.631*	.686*	.669*	.636*	.614*	.958*	1			
D	.712*	.682*	.638*	.692*	.660*	.629*	.627*	.969*	.902*	1		
A	.667*	.648*	.587*	.628*	.635*	.610*	.585*	.945*	.831*	.892*	1	
TI	-.647*	-.647*	-.586*	-.640*	-.580*	-.546*	-.496*	-.617*	-.599*	-.609*	-.565*	1

*P<0.01; OC, Perceived Organizational Climate; MS, Managerial Support; HRM, Human Resource Management; RS, Resource Support; TB, Team Behavior; QM, Quality Management; EBNS, Evidence-Based Nursing Support; WE, Work Engagement; V, Vigor; D, Dedication; A, Absorption; TI, Turnover Intention.

### 3.5 Analysis of the mediating effect of work engagement

First, the unmeasured latent method construct (UMLC) approach was employed to assess common method bias. A common method factor was added to the structural model, the absolute change of RMSEA was less than 0.05, and the absolute changes of GFI, CFI, NFI, IFI, TLI were all less than 0.1 (25). Therefore, common method bias was not a significant concern in this study.

Second, multicollinearity was assessed for the six dimensions of Perceived Organizational Climate. VIF values ranged from 3 to 8, while some exceeded 5, all remained below the threshold of 10 (29). This indicates that multicollinearity did not significantly threaten the stability of the SEM estimates.

**Figure 1:**
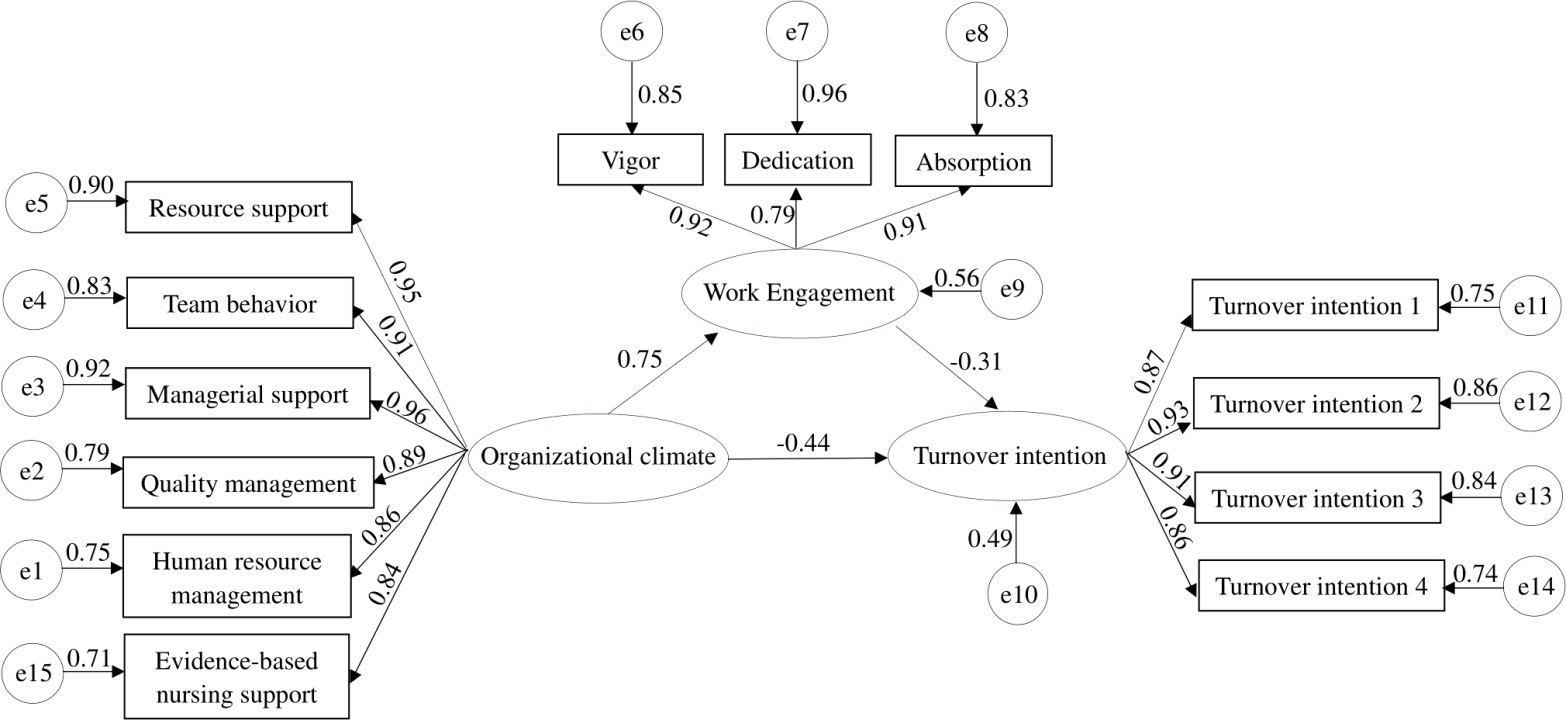
The hypothesized partial mediation model of work engagement in the relationship between perceived organizational climate and turnover intention.

Next, a mediation analysis was performed to examine the relationship among work engagement, perceived organizational climate, and turnover intention, with major demographic covariates controlled for. The results indicated that the mediating effect remained significant and the primary path coefficients were not substantially altered after adjusting for these variables. The final model demonstrated a good fit to the data, with the following fit indices: χ²/df = 2.849 (<3.0), GFI = 0.932, CFI = 0.981, NFI = 0.971, IFI = 0.981, TLI = 0.975, and RMSEA = 0.071 (<0.08). All indices met or exceeded the recommended criteria, indicating that the model was acceptable. The path diagram of the mediation model is shown in **Figure 1**.

Finally, a bias-corrected bootstrapping procedure (5,000 resamples) was used to test the significance of the mediating effect. As presented in **Table 5**, indicated that the Bootstrap 95% confidence interval for the indirect effect path did not include 0, confirming the presence of a significant mediating effect. Thus, work engagement partially mediated the association between perceived organizational climate and turnover intention, with the mediating effect accounted for 34.78% of the effect.

**Table 5:** Bootstrap analysis of the mediating effect.

Effect	Path	Effect value (β)	95% CI lower	95% CI upper	P-value	Effect proportion (%)
Indirect Effect	OC -> WE -> TI	-0.233	-0.344	-0.118	<0.001	34.78
Direct Effect	OC -> TI	-0.436	-0.584	-0.286	<0.001	65.22
Total Effect		-0.669	-0.735	-0.595	<0.001	100.00

OC, Perceived Organizational Climate; WE, Work Engagement; TI, Turnover Intention; CI, Confidence Interval.

## 4 Discussion and implications

### 4.1 Analysis of the current status of key variables among young nurses

The findings of this study indicate that the turnover intention among young nurses is at a moderately high level. This is consistent with recent research on similar populations in China (26), yet it appears to exceed levels reported among early-career nurses in some European countries (27). This disparity might be partially explained by the specific structural pressures within the Chinese “tertiary hospital” system, particularly the significantly higher patient-to-nurse ratios and intensive workload. Notably, items such as “I will leave the nursing profession as soon as I find another job” received high scores, suggesting that for many young nurses, turnover intention may have progressed from a passive psychological state to a proactive behavioral inclination. From a humanistic care perspective, this trend reflects a potential deficit in the emotional and professional support provided by organizations, failing to adequately meet the psychological needs of nurses at a critical career stage. According to the JD-R model, persistent job demands (e.g., high emotional labor, work-family conflict) could continuously deplete nurses’ psychological resources. For young nurses, who are in the early stages of their careers and simultaneously facing the pressures of establishing a family, making this resource depletion especially acute. Consequently, when perceived organizational support and humanistic care are insufficient to offset these heavy demands, they may be more inclined to contemplate alternative career paths as a self-protective coping mechanism (28).

Despite a positive overall perceived organizational climate, the “Resource Support” dimension yielded the lowest score. The deficiency in resource support reveals a structural bottleneck: for young nurses in their intensive professional formative stage, current compensation, promotion opportunities, and participation in decision-making fail to meet their high self-actualization needs. Compared to senior staff, young nurses often face more rigid career ladders and fewer opportunities for organizational recognition, which significantly deflates their perceived of organizational support. Interestingly, a paradoxical finding was observed where high team cohesion coexists with high turnover intention. Grounded in JD-R theory and humanistic care, while positive peer collaboration serves as a vital emotional buffer against daily stressors, it is sufficient to offset the “structural deficits” in resource support (29). In high-pressure nursing environments, strong team cohesion often emerges as a coping mechanism for survival (e.g., “mutual defense”). However, when young nurses envision their long-term future, the lack of career growth outweighs the emotional satisfaction derived from peer support. Essentially, they may “love their colleagues but leave the organization,” highlighting that interpersonal harmony cannot substitute for fundamental organizational care and professional advancement. Internationally, our findings align with Asian research but contrast with Western frameworks (30). For instance, the US “Magnet Hospital” model emphasizes that structural empowerment as a primary driver of retention (31). Unlike peers in Australia or Northern Europe who typically enjoy greater clinical autonomy and a significant “say” in decision-making (32), young nurses in our sample may feel constrained by top-down leadership. This cross-cultural variance highlights that while team synergy is a universal strength, the systemic empowerment of young nurses remains a critical challenge for humanistic nursing management globally.

Meanwhile, the work engagement of young nurses was at a moderately high level, similar to the findings of Poku et al. (33). From a humanistic perspective, this robust engagement is not merely a reaction to administrative policies, but reflects an internalized moral response to the professional mission. The participants, predominantly holding bachelor’s degrees or higher, likely foster a strong sense of professional identity, enabling them to reframe stressful clinical tasks as meaningful professional achievements (34). This internal cognitive process helps them maintain high engagement by focusing on professional fulfillment rather than just physical labor. However, the “Dedication” dimension scored relatively lower, revealing a critical gap that standard organizational support often overlooks. While traditional management may secure a nurse’s physical vigor and task-based absorption, it often fails to nurture the affective internalization required for nursing to be felt as a “calling” (35). This discrepancy suggests that current clinical environments may still treat nurses primarily as “care providers” rather than “recipients of care”. Adding the lens of humanistic care clarifies that without an organizational ethic of care, one that explicitly affirms a nurse’s professional worth and emotional needs, work engagement remains a fragile technical commitment rather than a resilient humanistic devotion, posing a latent risk for career instability (36).

### 4.2 Effects of perceived organizational climate and work engagement on turnover intention

This study also confirmed the significant correlations among perceived organizational climate, work engagement, and turnover intention. The strong correlation between perceived organizational climate and turnover intention indicates that favorable perceptions of organizational climate tend to align with lower turnover intentions within the young nurse cohort. These findings align with prior studies showing that a supportive, respectful, and fair organizational environment acts as a powerful “glue,” directly enhancing nurses’ sense of belonging and loyalty, thereby reducing their desire to leave (37, 38). Given their limited professional tenure, young nurses often exhibit a relatively weak sense of organizational belonging and identity. Since leadership is a primary determinant of a humanistic organizational climate, it is crucial for managers to embody caring and empathetic leadership to foster a nurturing work environment (39). Such human-centered support can effectively enhance nurses’ professional well-being and, ultimately, reduce turnover.

Work engagement is also recognized as an important correlate of turnover intention. Meta-analytic evidence indicates that the impact of work engagement on turnover intention is significantly more pronounced among nurses than in other occupational groups (40). The Ethics of Care theory provides a profound lens to interpret this finding: a positive organizational climate creates a sense of psychological safety and mutual respect, which is linked to enhanced work engagement (13). This environment encourages nurses to adopt adaptive cognitive emotion regulation strategies, helping them not just to perform a role, but to find meaning and professional dignity within their practice, ultimately reflecting a lower inclination to leave (41). Consequently, managers should adopt supportive styles, such as caring, ethical, or transformational leadership, by implementing structured mentorship that provides young nurses with emotional “buffering” alongside professional guidance. By fostering a “just culture” and emphasizing team interconnectedness, organizations can create a nurturing environment that recognizes individual value, thereby bolstering work engagement and effectively mitigating turnover intention.

### 4.3 The mediating role of work engagement

The core finding of this study confirms the partial mediating role of work engagement in the relationship between perceived organizational climate and turnover intention(indirect effect = 34.78%). This result extends the motivational process of the JD-R model by revealing the “how”: a positive clinical climate, particularly one imbued with humanistic care, functions as a high-quality job resource that fulfills young nurses’ basic psychological needs for autonomy and relatedness (42). Unlike general organizational support, a humanistic climate, characterized by recognition and emotional support, serves as a catalyst that transforms external resources into internal vigor and dedication. According to Kahn’s theory of engagement (43), young nurses are more willing to invest their personal energy into their work roles when they feel psychologically safe, find their work meaningful, and feel available. High levels of work engagement are not just a positive state but also a valuable psychological resource that helps nurses cope more effectively with job stress.

Notably, the partial nature of this mediation suggests that work engagement is not the sole driver of retention. From the perspective of Social Exchange Theory (44), a supportive climate may also directly foster organizational commitment or enhance professional identity, which could independently reduce turnover intention regardless of the level of engagement. Furthermore, the effectiveness of this pathway likely varies depending on individual or situational characteristics. For instance, the impact of organizational climate may be more critical for young nurses with lower psychological resilience. For young nurses early in their careers, managerial and resource support serve as primary buffers against turnover intention. By acknowledging these specific dimensions and individual differences, this study provides a more nuanced framework, emphasizing that “caring for the caregivers” requires tailored strategies to maximize the retention of the nursing workforce.

### 4.4 Managerial implications from a humanistic care perspective

The results of this study offer significant managerial implications. From a humanistic care perspective, the key to reducing turnover among young nurses lies in translating the abstract concept of “care” into tangible organizational behaviors and institutional support.

Given that “Resource Guarantee” scored the lowest in our assessment, cultivating a caring ethical climate is a vital strategy. In such a climate, the hospital upholds altruistic principles and cares for every medical staff member, recognizing that nurses also require support due to their professional vulnerability. Managers should integrate care into tangible institutional behaviors and regulations, such as optimizing workflows through information technology (e.g., smart nursing systems and simplified documentation) to reduce burdens and increase efficiency. By providing necessary resources and fostering a “no-blame” environment, administrators can create a stable support system that directly alleviates turnover pressure.

The 34.78% partial mediation of work engagement reveals that nursing management should also focus on “igniting the intrinsic work passion” of young nurses, especially since our data showed that “Dedication” was their least-scoring engagement dimension. To address this, hospitals should formulate caring-based policies that empower young nurses at the career development level. Instead of top-down supervision, managers should adopt “caring leadership” to proactively listen to their needs and pay attention to work-life balance. Crucially, by designing clear career ladders and involving young nurses in departmental decision-making (shared governance), hospitals can enhance their sense of professional dignity and ownership. Integrating care into such policies sends a powerful signal that the organization values their long-term development, effectively bridging a positive organizational climate with their professional dedication and intention to stay.

## 5 Conclusion and limitations

This study identifies significant interconnections among perceived organizational climate, work engagement, and turnover intention among young nurses, with work engagement representing a critical intermediary mechanism. Theoretically, these findings empirical validate the JD-R motivational pathway within a reciprocal humanistic framework, demonstrating that an organizational climate rooted in care functions as a vital job resource. While the cross-sectional nature of this research limits causal inferences, the results suggest that addressing turnover requires a paradigm shift toward caring-based policies that uphold altruistic principles and protect the interests of healthcare staff. By integrating structural support with a supportive ethical environment, healthcare institutions can effectively translate organizational care into professional vitality, ensuring the long-term stability of the young nursing workforce.

There are certain limitations of this study. First, the cross-sectional design precludes causal inferences. Future longitudinal designs are needed to track these variables over time and establish causality. Second, the reliance on convenience sampling from a single tertiary hospital may introduce selection bias, thereby limiting the representativeness and generalizability of the findings to other hospital types or regions. Third, all data were collected via self-report questionnaires, which may be subject to common method bias. While we employed the Unmeasured Latent Method Construct (UMLC) approach to rigorously assess common method bias, it cannot fully eliminate the subjectivity of self-reporting. Finally, although major demographic and professional factors were controlled for in our analysis, the potential for omitted variable bias cannot be entirely ruled out. Future studies should prioritize multi-site and cross-cultural comparisons to test the model’s universality. Furthermore, incorporating additional mediators or moderators, such as psychological resilience or professional identity, alongside multi-source data (e.g., peer or supervisor ratings), would provide a more comprehensive and rigorous understanding of the mechanisms driving nurse retention.

## Data Availability

The raw data supporting the conclusions of this article will be made available by the authors, without undue reservation.
